# Inferring gene expression dynamics via functional regression analysis

**DOI:** 10.1186/1471-2105-9-60

**Published:** 2008-01-28

**Authors:** Hans-Georg Müller, Jeng-Min Chiou, Xiaoyan Leng

**Affiliations:** 1 Department of Statistics, University of California, One Shields Avenue, Davis, CA 95616, USA; 2 Institute of Statistical Science, Academia Sinica, Taipei 115, Taiwan; 3 Wake Forest University School of Medicine, Division of Public Health Sciences, Department of Biostatistical Sciences, One Medical Center Blvd., Winston-Salem, NC 27157, USA

## Abstract

**Background:**

Temporal gene expression profiles characterize the time-dynamics of expression of specific genes and are increasingly collected in current gene expression experiments. In the analysis of experiments where gene expression is obtained over the life cycle, it is of interest to relate temporal patterns of gene expression associated with different developmental stages to each other to study patterns of long-term developmental gene regulation. We use tools from functional data analysis to study dynamic changes by relating temporal gene expression profiles of different developmental stages to each other.

**Results:**

We demonstrate that functional regression methodology can pinpoint relationships that exist between temporary gene expression profiles for different life cycle phases and incorporates dimension reduction as needed for these high-dimensional data. By applying these tools, gene expression profiles for pupa and adult phases are found to be strongly related to the profiles of the same genes obtained during the embryo phase. Moreover, one can distinguish between gene groups that exhibit relationships with positive and others with negative associations between later life and embryonal expression profiles. Specifically, we find a positive relationship in expression for muscle development related genes, and a negative relationship for strictly maternal genes for *Drosophila*, using temporal gene expression profiles.

**Conclusion:**

Our findings point to specific reactivation patterns of gene expression during the *Drosophila *life cycle which differ in characteristic ways between various gene groups. Functional regression emerges as a useful tool for relating gene expression patterns from different developmental stages, and avoids the problems with large numbers of parameters and multiple testing that affect alternative approaches.

## Background

### Biological motivation and overview

Normal development of an organism depends on precisely regulated temporal and spatial expression of its genes. In unicellular organisms, such as yeast, different sets of genes are expressed at different stages of the cell cycle. In higher organisms, with very few exceptions, all of the different types of cell possess the same genes; however each type of cell only expresses a unique set of "signature" genes at a certain time, depending on current developmental tasks [1]. Different life stages of an organism are thought to share the same or similar set of "signature" genes, which thus play a role throughout ontogenesis. For example, there are two phases of somatic muscle formation in the development of *Drosophila melanogaster*. The first phase of myogenesis occurs during embryonic development and generates larval muscle elements that mediate the relatively simple behaviors of the larva. During pupal metamorphosis, a second phase of myogenesis generates a diverse pattern of muscle fibers, facilitating the more complex behaviors of the adult fly [2].

While appreciating intrinsic differences between these two phases of myogenesis in *Drosophila*, it seems plausible that the genes involved in embryonal myogenesis are re-activated, perhaps in a modified way, during the assembly of adult muscles [3]. In a recent study of temporal gene expression during the life cycle of *Drosophila *by Arbeitman et al., using cDNA microarrays, a group of "muscle" genes was identified from gene expression time courses [4]. Viewed over the entire life cycle, genes in this group exhibit a two-peak expression pattern where the timing of expression peaks coincides with the timing of larval and adult muscle development. Another example is provided by the group of "strictly maternal" genes of *Drosophila*. It has been suggested that "if the information to build the fly is already deployed in the newly laid egg, then the genes that encode that information must be expressed and utilized while the egg is being constructed, during oogenesis" [5]. This was confirmed by Arbeitman et al., who showed that the "strictly maternal" genes had peaks during the very early hours of embryonal development and were only re-expressed at higher levels in the female germline during oogenesis [4].

It seems straightforward that within a specific biological pathway a gene with higher expression level in an early stage of development also tends to be expressed more in a later stage. However, to the best of our knowledge, this issue has not been quantitatively investigated in the temporal gene expression framework. It is therefore of interest to quantify repeated patterns of gene activation over the life cycle. Such patterns may reflect properties of the molecular basis of events during ontogenesis that underlie the cellular changes. On the other hand, as the same set of "signature" genes may appear at different life stages, they orchestrate similar yet distinct phases of development. There appear to be two levels of genetic information being accessed during the different phases: the common information of the same development and the specialized information pursuant to the distinctive phases. This motivates the task to quantitatively ascertain relationships of gene expression trajectories for various developmental stages. Variation in the patterns of gene expression may point to unique morphological, physiological or molecular properties of individual phases that manifest themselves in phase-gene specific interactions.

To study these questions, we take advantage of the temporal microarray gene expression data collected by Arbeitman et al. for *Drosophila*, with the specific goal to regress later life gene expression patterns on those of embryonal gene expression [4]. Since these expression patterns are time-dynamic, the goal of relating various gene expression dynamics to each other requires the deployment of statistical methodologies that are adequate for regressing time courses on each other. Functional regression analysis is a promising tool to ascertain such relationships. The ultimate goal is to gain insights into pathways that are repeatedly activated during the life cycle. We demonstrate here that distinct relationships exist between later life expression trajectories and embryonal trajectories, which can be summarized as positive and negative association. Positive association describes groups of genes for which higher embryonal expression tends to be followed by higher expression during reactivation of these genes in later life as well, with opposite effects in the case of negative association.

### Temporal gene expression profiles for the *Drosophila *life cycle

**Figure 1:**
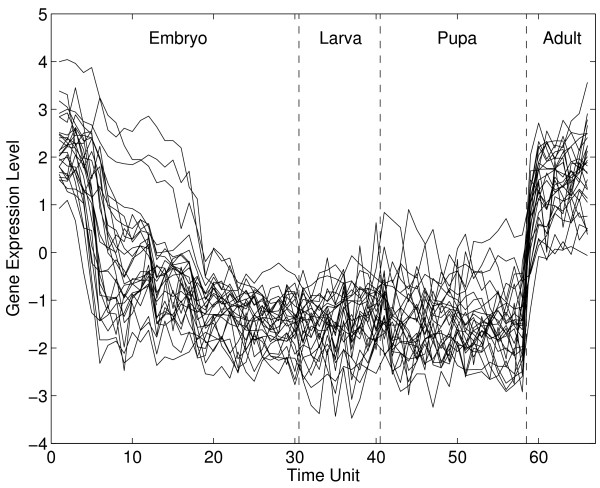
A subset of observed gene expression profiles (strict maternal genes). Each profile (or curve) is composed of expression levels of one gene at different time points.

Arbeitman et al. reported cDNA microarray transcriptional profiles for nearly one-third of all 4028 Drosophila melanogaster genes throughout a complete life cycle, covering 66 sequential time points, beginning at fertilization and spanning the embryonic, larval and pupal periods and the first 30 days of adulthood, when males and females were sampled separately (Fig. 1) [4]. Groups (or clusters) of co-expressed genes were identified. In this paper we study the nature of dynamic relationships of expression during different life cycle phases for two groups of such genes.

A first group is composed of 23 "muscle" genes. The tissue-specific (expressed in muscle tissue) genes in this group have a two-peak expression pattern that coincides with larval and adult muscle development. Larval muscle development is initiated in the embryo by the gene *twist *(*twi*), which directly regulates another gene *dMef2*. Some *twi*-expressing cells are set aside during embryonic myogenesis to contribute to adult-specific muscle formation. In both larval and adult muscle development, *dMef2 *is required for the differentiation of the various muscle types [3,6]. Fifteen of the 23 genes in this group (65%) contain pairs of predicted *dMef2*-binding sites, so that many of the genes in this group are likely to be direct targets of *dMef2 *[4]. Our goal is to study the dependence of adult gene expression patterns on larval patterns for this group of genes.

A second group of genes that we consider are 27 "strictly" maternal genes. These maternal genes are responsible for the polarity of the egg and ultimately, the embryo. Each of these is deposited into the egg during oogenesis by the mother prior to fertilization, in preparation for later function during embryonic development. Transcripts from all these genes are degraded after fertilization and are not re-expressed until oogenesis in the female germline [4]. Our interest is to assess gene expression pattern dependencies between early embryo and the female adult germline for this group of genes.

### Key features and relevance of functional regression

The developmental gene-specific expression time courses are viewed as being generated by an underlying smooth random trajectory which is specific to each gene. These trajectories are sampled at a grid of measurement points during each life cycle phase, e.g., *s*_1_,...,*s*_*p*_, where *p *= 31 for the measurements of gene expression during the embryonal period. If we denote the embryonal phase predictor trajectories by *X*_*i*_(*s*), where *i *is a gene index, then the observed data for the embryonal gene expression are *X*_*ij *_= *X*_*i*_(*s*_*ij*_) + *e*_*ij*_, where the *e*_*ij *_are measurement errors which are assumed to be independent, with zero mean and finite variance.

The situation is analogous for the response trajectories *Y*_*i*_(*t*) which pertain to measured expression of the same genes but for a different developmental phase and also give rise to a set of analogously defined discrete measurements (*t*_*ik*_, *Y*_*ik*_). As the gene expression dynamics in our model is reflected by the entire embryonal and later life trajectories, the task arises to relate response trajectories *Y*_*i *_to predictor trajectories *X*_*i*_. A basic problem here is the high dimension of both responses and predictors. As further detailed in Section 4.1, classical regression models do not provide good estimation for this situation and classical regression inference is hampered by the high dimensions and the necessity to adopt multiple testing procedures. Functional regression on the other hand incorporates an automatic dimension reduction step which is data-adaptive and therefore estimation relies on only few parameter estimates. An overall functional coefficient of determination *R*^2 ^in conjunction with the bootstrap can be used for functional inference.

Functional linear regression, where both predictors and responses are trajectories, can be understood as an extension of multivariate linear regression. The development of functional regression falls within the expanding area of functional data analysis [7]. Recent work for the case where predictors are scalars and the responses are trajectories includes references [8-11]. For the purpose of relating gene expression trajectories to each other, a functional regression setting in which both predictor and response are trajectories is appropriate [12,13].

We extend previous methodological work in various directions useful for the analysis of gene expression trajectories, demonstrating the following beneficial features: (1) functional linear regression can be broken down into a series of linear regressions of functional principal component scores of the response trajectories on those of the predictor trajectories; (2) this decomposition leads to a straightforward implementation of functional regression via a series of simple linear regressions; (3) the decomposition opens up alternative ways to interpret a functional regression relation; (4) outliers and influential trajectories corresponding to individual genes can be identified with this methodology; and (5) inference for functional regression can be obtained via bootstrapping.

In biological applications of functional regression it is often of primary interest to test whether a functional regression relationship exists for given data. For this objective, the proposed bootstrap method is very useful; besides testing for significance of a functional regression, it can also be used to construct confidence regions. Equally important is the interpretability of the results, for which the decomposition into simple linear regressions provides an useful alternative. For the developmental *Drosophila *gene expression profiles, various types of dependency of adult on embryonal gene expression trajectories can be clearly distinguished through the application of these functional regression tools, reflecting differences in the underlying dynamics of gene expression. The proposed methodology is inherently nonparametric and therefore very versatile.

## Methods

The description of the methods in the following includes some technical material that is not essential for the description of the results in the following sections but is provided so as to give a complete account of the functional regression methodology. The methodology described below and used for the analysis of gene expression trajectories has been implemented in Matlab and is freely available in the PACE (principal analysis by conditional expectation) package, downloadable from the internet [14].

In this program, if one chooses default values, the only required input for the functional regression part PACE-REG are the measurements of gene expressions for predictor and response trajectories, denoted in the previous section by (*s*_*ij*_, *X*_*ij*_) and (*t*_*ik*_, *Y*_*ik*_) where *i *ranges over the genes, *j *over the predictor measurements (its range may depend on *i*), and *k *over the response measurements (range may depend on *i*). As outputs one obtains the quantities as shown in the results section.

### Preliminaries on functional linear regression

Denoting the random predictor and response functions by *X*(*s*) resp. *Y*(*t*), their mean functions by *μ*_*X*_(*s*) = *E*(*X*(*s*)), *μ*_*Y *_(*t*) = *E*(*Y*(*t*)) and their covariance functions by *G*_*X*_(*s*_1_, *s*_2_) = cov(*X*(*s*_1_), *X*(*s*_2_)), *G*_*Y *_(*t*_1_, *t*_2_) = cov(*Y*(*t*_1_), *Y*(*t*_2_)), one obtains under mild conditions the Karhunen-Loève expansions for trajectories *X *and *Y*, given by

(1) $$X(s)=\mu_{X}(s)+\sum_{j=1}^{\infty }\xi_{k}\varphi_{k}(s),$$

(2) $$Y(t)=\mu_{Y}(t)+\sum_{k=1}^{\infty }\zeta_{k}\psi_{k}(t)$$

[15, see Appendix for further explanations]. These representations provide a convenient way to implement the necessary dimension reduction for the trajectories *X *and *Y*, by truncating the sums on the r.h.s. at a finite number of terms, where the truncation point needs to be chosen data-adaptively (flatter and simpler structured trajectories requiring fewer and more complex trajectories requiring more components to be included). The trajectories are represented by their overall mean function, random coefficients *ξ*_*j *_resp. *ζ*_*k *_and the sequence of basis functions *φ*_*j *_resp. *ψ*_*k*_, with indices *j *and *k *ranging between 1 and the truncation value, say 1 ≤ *j *≤ *J *for *X *and 1 ≤ *k *≤ *K *for *Y*. The functions *φ*_*j *_and *ψ*_*k *_are chosen as eigenfunctions and are often referred to as "modes of variation": They represent the main "directions" in function space in which the trajectories vary. Representations (1) and (2) are analogous to expressing a centered random vector in terms of the basis of the vector space that consists of the eigenvectors. The random effects *ξ*_*j*_, *ζ*_*k *_are centered at zero and are referred to as functional principal component scores, or just scores. Additional mathematical details can be found in the Appendix.

The functional linear regression model with response function *Y *and predictor function *X *is

(3) $$E(Y(t)|X)=\mu_{Y}(t)+\int_{S}\beta (s,t)(X(s)-\mu_{X}(s))ds,$$

where the bivariate regression parameter function *β*(*s*, *t*) is smooth and square integrable [12]. This model emerges as a generalization of the multivariate linear regression model *E*(*Y*|*X*) = *BX*, where *X *and *Y *are random vectors and *B *is a parameter matrix. The function *β *is central to this functional regression model. For fixed *t*, the value of the response trajectory at *t*, which is *Y*(*t*), is obtained by integrating the predictor trajectory over its domain with the weight function *β*(*s*, *t*), viewed as a function of *s *for fixed *t*.

One way to interpret the functional regression is therefore to fix various levels of *t *and to then inspect these weight functions by taking the appropriate cross-section through the surface *β*(*s*, *t*) when *t *is held fixed at the selected level. This weight function then indicates which parts of the predictor trajectory contribute positively or negatively to the outcome *Y*(*t*). Under regularity assumptions, the regression parameter surface *β *has the following basis representation,

(4) $$\begin{array}{ll} \beta (s,t)=\sum_{k=1}^{\infty }\sum_{j=1}^{\infty }\beta_{kj}\varphi_{j}(s)\psi_{k}(t), & \beta_{kj}=\frac{E(\xi_{j}\zeta_{k})}{E(\xi_{j}^{2})}. \end{array}$$

A detailed description of how the mean functions *μ*_*X*_, *μ*_*Y*_, eigenfunctions *φ*_*j*_, *ψ*_*k *_and eigenvalues *λ*_*j*_, *τ*_*k *_can be consistently estimated from noisy data by using nonparametric smoothing methods can be found in references [13,16]. These estimation steps involve pooling the measurements for all predictor (resp. response) trajectories and then applying a smoothing method to obtain the mean function *μ*_*X*_, and to smooth pointwise "raw" covariances (omitting the diagonal variances) to obtain the smooth covariance function cov(*X*(*s*_1_), *X*(*s*_2_)). Then eigenfunctions are obtained by numerical discretization and spectral decomposition of the resulting covariance matrix and projected onto a positive definite covariance function. This leads to estimates ${\hat{\xi }}_{j}$, ${\hat{\zeta }}_{k}$ of the functional principal component scores *ξ*_*j*_, *ζ*_*k*_. These estimates must cope with noise in the observed expression data and therefore are implemented as estimated conditional expectations. The resulting estimates are then plugged in on the r.h.s. of eq. (4) to obtain an estimate of the regression parameter function *β*.

### Decomposing functional linear regression into simple linear components

As is shown in the Appendix, the relationships between response scores within the functional linear model are simple: They are simple linear regressions through the origin, i.e.,

(5) *E*(*ζ*_*k*_|*ξ*_*j*_) = *β*_*kj*_*ξ*_*j*_.

Here the slope coefficients *β*_*kj *_of these simple linear regressions define the functional regression parameter function according to (4). This leads to the conclusion that functional linear regression can be decomposed into a series of simple linear regressions of the functional principal component scores of response processes on those of predictor processes.

This fact substantially simplifies the analysis and provides an alternative interpretation of the resulting functional linear regression model. Note that representation (4) implies that for given slopes *β*_*kj*_, the regression parameter surface *β*(·,·) is uniquely determined. It follows from (12) in the Appendix that the inverse also holds. Therefore, a functional linear relationship between random trajectories *Y *and *X *can be described equivalently by the regression parameter function *β*(*s*, *t*) or the doubly-indexed sequence of all slope coefficients *β*_*kj*_, *k*, *j *≥ 1.

A consequence of this equivalence is that estimates of the slope coefficients can be directly used to obtain straightforward estimates of the regression parameter function. Another consequence of this equivalence is that it opens up two different perspectives for the interpretation of a functional linear regression relation: Either through features of the shape of the regression parameter surface *β*(*s*, *t*), or through the ensemble of the regression slopes *β*_*kj*_, *k*, *j *= 1, 2,.... For the latter, the interpretation is in terms of changes towards the direction of the corresponding eigenfunctions. For example, if the slope coefficient *β*_11 _relating the first predictor score to the first response score is positive and large, it implies that as the predictor trajectories move increasingly towards the direction marked by *φ*_1_, i.e., from its mean *μ*_*X *_towards *μ*_*X *_+ *γφ*_1 _for increasing *γ*, response trajectories move increasingly towards direction *ψ*_1_, i.e., from *μ*_*Y *_towards *μ*_*Y *_+ *γψ*_1 _for increasing *γ*. The other slope coefficients can be interpreted analogously.

Often there will be interest in measuring the strength of association between predictors and responses in a functional regression. The classical measure in a multiple linear regression relationship is the coefficient of determination *R*^2^. Extensions to the functional case were discussed in reference [13]. When applying the decomposition into simple linear regressions, this functional coefficient of determination assumes a particularly simple form.

### Implementing the decomposition

Given estimates ${\hat{\varphi }}_{j}$, ${\hat{\psi }}_{k}$, ${\hat{\xi }}_{ij}$ and ${\hat{\zeta }}_{ik}$ of eigenfunctions and functional principal component scores, the regression parameter surface is estimated by

(6) $$\hat{\beta }(s,t)=\sum_{k=1}^{K}\sum_{j=1}^{J}{\hat{\beta }}_{kj}{\hat{\varphi }}_{j}(s){\hat{\psi }}_{k}(t),$$

where unknown slope estimates ${\hat{\beta }}_{kj}$ can be obtained by least squares estimation of the slopes in scatterplots {${\hat{\zeta }}_{ik}$} on {${\hat{\xi }}_{ij}$}, fitted without intercept, for all *k *= 1,..., *K*, and *j *= 1,..., *J*. These least squares estimates are however subject to attenuation and therefore bias due to the presence of errors in the predictors {${\hat{\xi }}_{ij}$} (corresponding to errors-in-variables), as these need to be estimated from the data and are therefore imprecise. An alternative estimate that is less subject to this problem is given by

(7) $$\begin{array}{cc} {\hat{\beta }}_{kj}=\frac{1}{(n-1){\hat{\lambda }}_{j}}\sum_{i=1}^{n}\left({\hat{\xi }}_{ij}-\bar{\hat{\xi }}._{j})({\hat{\zeta }}_{ik}-\bar{\hat{\zeta }}._{k}\right), & 1\leq j\leq J,\;1\leq k\leq K, \end{array}$$

as the denominator is estimated directly from the covariance surface where the measurement errors are confined to the diagonal which allows to eliminate their effect to a large extent. If the denominator is estimated from empirically observed functional principal component scores, as is the case in the usual least squares estimators, then contamination by error may lead to inflated empirical variances, and as a result to attenuation of the estimated regression coefficients. In the following we therefore adopt the alternative estimate (7).

Note that *J *and *K *denote the numbers of random components of predictor resp. response processes to be included in the functional regression analysis. These numbers can be determined in practice by scree plots, displaying the fraction of variance explained as the number of included components increases. This simple and fast approach leads to adequate results, choosing the smallest number of components that explain 85% of the variation. Alternative selectors are discussed in references [13,17]. Based on (13), we insert the chosen values of *J *and *K *to obtain the estimated coefficient of determination

(8) $$\begin{array}{ccc} {\hat{R}}^{2}=\sum_{j=1}^{J}\frac{\sum_{k=1}^{K}{\hat{R}}_{kj}^{2}{\hat{\tau }}_{k}}{\sum_{k=1}^{K}{\hat{\tau }}_{k}}, & \text{with} & {\hat{R}}_{kj}^{2}=\frac{{\hat{\beta }}_{kj}^{2}{\hat{\lambda }}_{j}}{\tau_{k}}, \end{array}$$

using estimates (7). Here the ${\hat{R}}_{kj}^{2}$ are the estimated coefficients of determination of the simple linear regressions of *ζ*_*ik *_on *ξ*_*ij*_, which target (14).

### Bootstrap inference

Inference for the functional regression that overcomes the high dimension and multiple testing problem is obtained by the bootstrap. In the face of the complexity of the dependencies between estimated functional principal component scores and estimated eigenfunctions, the regression bootstrap with resampling from the sample of all pairs of predictor and response trajectories emerges as a doable approach. The starting point for generating the bootstrap samples is to randomly sample *n *units from trajectory indices {1, 2,..., *n*} by sampling with replacement and, for each sampled index i*, entering all observations for the corresponding predictor and response trajectories. This sampling procedure is repeated until *B *bootstrap samples, consisting of predictor and response data for each of *n *trajectories, have been assembled.

For each of these bootstrap samples, we then carry out the functional linear regression procedure, obtaining all relevant estimates. The resulting estimates from the *B *bootstrap samples are used to construct pointwise confidence intervals for the regression parameter function *β*(·,·), simply by locating the corresponding lower and upper quantiles in the bootstrap distribution of the estimated surface values $\hat{\beta }$(*s*, *t*) (6) for all fixed *s*, *t*. The resulting upper and lower confidence surfaces will provide an idea how well the surface is actually determined from the data. Analogously, local confidence bands can be constructed for individual predicted trajectories, given any predictor function.

In addition to confidence intervals, it is a common objective in regression analysis to establish the "significance of the regression relation", i.e., to infer whether within the assumed model the predictors indeed influence the responses. The classical test for a multiple regression corresponds to the null hypothesis that all regression slope coefficients vanish, or equivalently, that the coefficient of determination *R*^2 ^= var(*E*(*Y*|*X*))*/*var(*Y*) vanishes. The extension to the functional case is not straightforward. In a first attempt to assess the overall significance of the functional regression, one can use pointwise bootstrap confidence intervals, say at the 95% level, and check whether there are areas where 0 is not included in these intervals. However, this does not properly account for the multiple confidence statements that are made. To obtain an overall bootstrap test for the significance of the functional regression relation, we use an alternative resampling procedure where predictors and responses are resampled separately under the null hypothesis of no regression relationship. For each resample, the data for a predictor trajectory and the data for a response trajectory are selected separately by sampling with replacement from all predictor and all response trajectories. As predictors and responses are unrelated in this case, applying functional regression to *B *such null bootstrap samples (each of size *n*) provides a null distribution for the pivotal statistic.

In our approach we specifically select the functional coefficient of determination *R*^2 ^(13) as test statistic, as it summarizes the regression effect. Accordingly, we obtain the overall *p*-value of the functional regression by determining the empirical quantile of the observed value of *R*^2 ^within the null bootstrap distribution for *R*^2^. Bootstrap inference for the overall regression effect based on this device will be illustrated in the application to gene expression profiles in the next section.

## Results

### Regressing pupa-adult temporal gene expression profiles on embryonal profiles for muscle-specific genes

**Figure 2:**
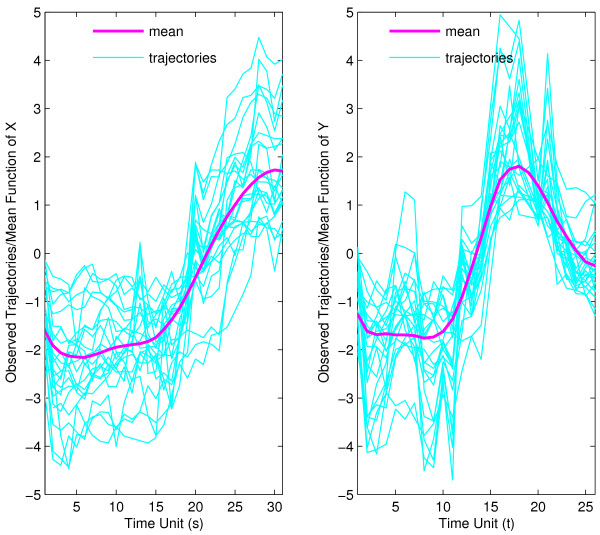
Observed trajectories and estimated mean function for muscle-specific genes for predictor profiles *X *(corresponding to gene expression profiles in embryo phase, left panel) and for response profiles *Y *(profiles for pupa-adult phase, right panel.

The functional response is the combined pupa-adult temporal gene expression profile, which we view as a smooth random function. For each of the 23 muscle-specific genes, these expression profiles are sampled at 26 time points during the combined pupa-adult phase. Likewise, the functional predictors are smooth random functions that correspond to the embryonal gene expression profiles, recorded for the same genes as the functional responses, and sampled at 31 time points. An initial step is the estimation of the mean trajectories for predictor and response. Along with the individual temporal gene expression profiles, these mean trajectories are shown in Fig. 2. They are flat in the beginning for both predictors and responses and for predictors increase monotonously in the right half, while they have a marked peak in the right half for the responses. This provides a general timeline for the expression of these muscle-specific genes.

**Figure 3:**
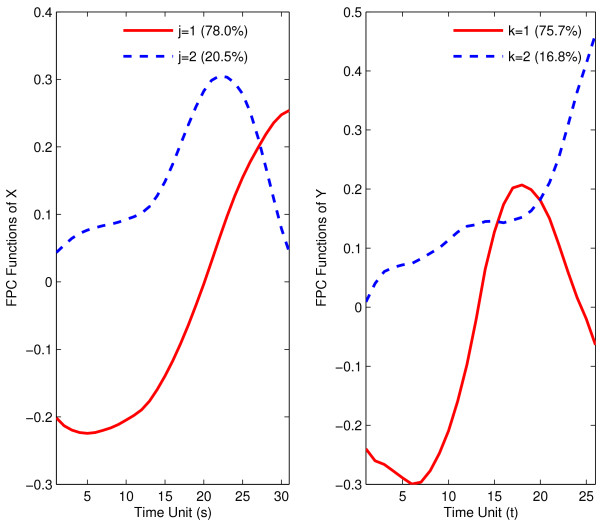
First two estimated eigenfunctions for temporal gene expression trajectories for the muscle-specific genes in embryo phase (predictors *X*, left panel) and pupa-adult phase (responses *Y*, right panel).

In a next step, implementing the main dimension reduction step, we select the first two eigenfunctions, separately for predictor and response trajectories; these are shown in Fig. 3. We find that the first eigenfunctions for both predictors and responses are roughly proportional to the mean function of Fig. 2. They explain 75–80% of total variation; the proportionality of mean function and first eigenfunction as seen here is commonly observed in functional data analysis (see the discussion of this phenomenon in reference [18]). The second eigenfunctions are restricted to be orthogonal to the first eigenfunctions and are found to not change sign in this application. For predictor processes, the first two eigenfunctions together explain 98.5% of total variation, while the corresponding figure is 92.5% for response processes.

**Figure 4:**
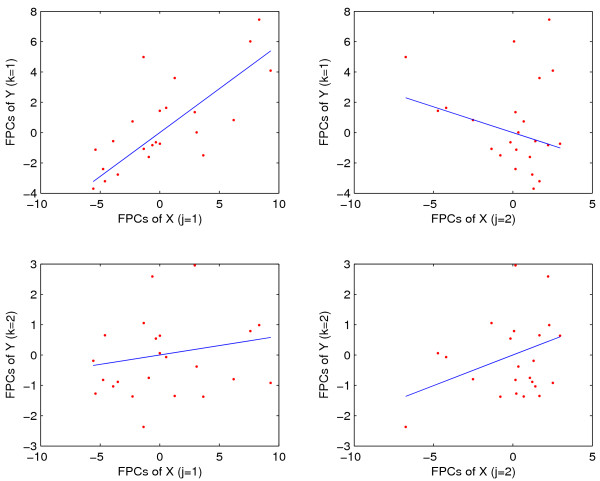
Scatterplots of functional principal component scores *ζ*_*k *_of response trajectories versus *ξ*_*j *_of predictor trajectories, for *j*, *k *= 1, 2, for muscle-specific genes with embryo phase as predictors *X *and pupa-adult phase as responses *Y*. Superimposed are simple linear regression lines, fitted without intercept. The functional coefficient of determination *R*^2 ^is 0.8479, with the bootstrap *p*-value *p *= 0.0010.

Decomposing the functional linear regression into simple linear components as described in Methods leads to four scatterplots visualizing first and second functional principal component scores of response processes versus first and second functional principal scores of predictor processes. These are shown in Fig. 4, along with the fitted least squares regression lines. We note that the positive slope of the simple linear regression of the first functional principal component scores on each other stands out (upper left panel of Fig. 4).

The functional coefficient of determination as defined in (13) is found to be *R*^2 ^= 0.85, with *p *value *p *= 0.0010, obtained via the null bootstrap (see Methods) and based on *B *= 1000 bootstrap samples. We note that the size of this functional coefficient of determination points towards a strong regression relationship. The associated bootstrap *p*-value is correspondingly small, indicating the significance of this functional regression relation. We may infer that as the expression profiles of predictor trajectories are increasing proportionally in the direction of the first eigenfunction, the response trajectory gene expressions also tend to increase in the direction of their first eigenfunction. Since the first eigenfunctions are overall quite similar to the mean expression trajectories, and explain most of the variation, another way to express this is to say that as the level of gene expression increases proportionally to the mean expression trajectories for embryonal muscle-specific genes, the expression of pupa-adult trajectories also tends to increase proportionally to their respective mean response trajectory.

**Figure 5:**
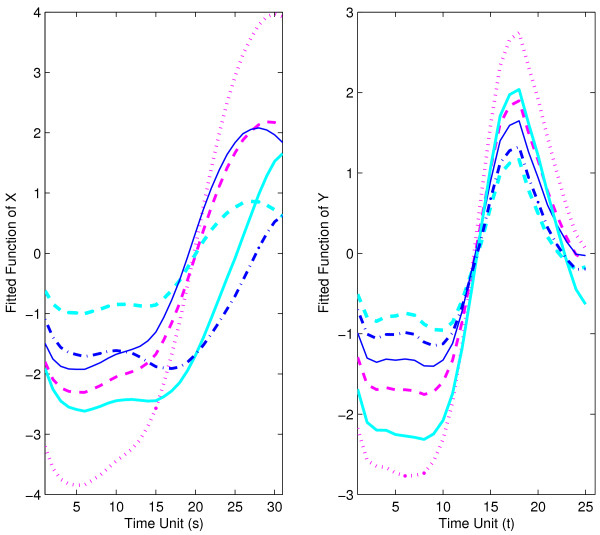
Predictor trajectories (left panel) and fitted response trajectories (right panel) for six randomly selected muscle-specific genes.

We conclude that for muscle-specific genes the expression patterns of both embryonal and pupa-adult phases are positively coupled in a relatively straightforward fashion. One could summarize the situation as proportionality of the activity patterns for muscle genes between adult and the embryo phases. This is an instance of functional proportionality. This functional proportionality with its positive coupling is also evident when looking at individual predictor trajectories and their associated fitted response trajectories; these are shown for a sample of randomly selected six genes in Fig. 5. The higher the amplitude of a predictor trajectory is, with more expressed features such as lower trough, steeper rise and higher peak, the more expressed are the features of the corresponding response trajectory, such as a higher peak.

### Functional linear regression of pupa-adult profiles on embryonal profiles for strict maternal genes

A functional regression relationship that clearly differs from the functional proportionality that was seen to be present for the muscle-related genes is found for the group of 27 strict maternal genes. The number of time points at which expression is available is the same as for the muscle-specific genes, with expression during the embryonal phase as predictor and expression during pupa-adult phase as response.

**Figure 6:**
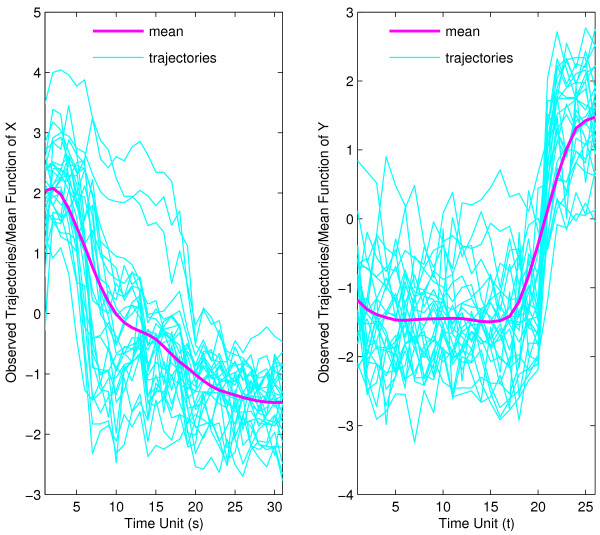
Observed trajectories and estimated mean function for strict maternal genes in embryo phase (for predictor *X*, left panel) and pupa-adult phase (for response *Y*, right panel), respectively.

As in the analysis for muscle-specific genes, we first plot individual gene expression trajectories for the embryonal predictor phase and the pupa-adult response phase overlaid with the mean trajectories in Fig. 6. We find distinct patterns of expression for both embryonal and pupa-adult phases, with fast early and then slower late declines in mean gene expression for the embryonal phase, while the mean trajectory for the pupa-adult phase shows low and nearly constant expression on average for the first 15 time units (the pupa phase), later followed by rapidly increasing expression (in the adult phase).

**Figure 7:**
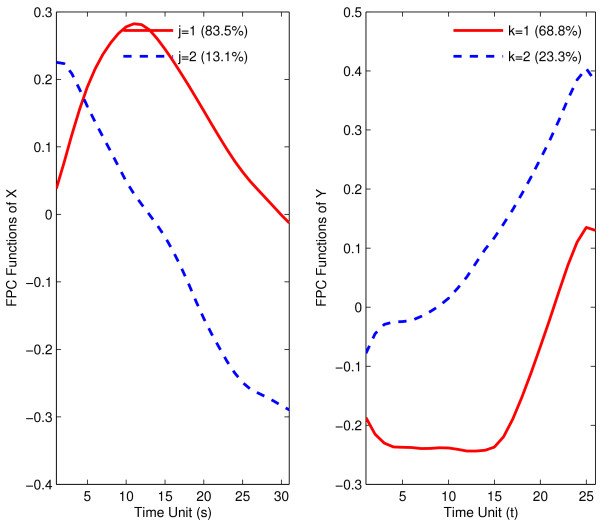
First two estimated eigenfunctions for strict maternal genes in embryo phase (predictor *X*, left panel) and pupa-adult phase (response *Y*, right panel).

Again, two eigenfunctions are chosen for response as well as predictor trajectories, shown in Fig. 7. In contrast to the muscle-specific genes, the first eigenfunction is not proportional to the mean function for predictor trajectories, while the second eigenfunction has this property. For response trajectories, the first eigenfunction is approximately proportional to the mean expression function.

**Figure 8:**
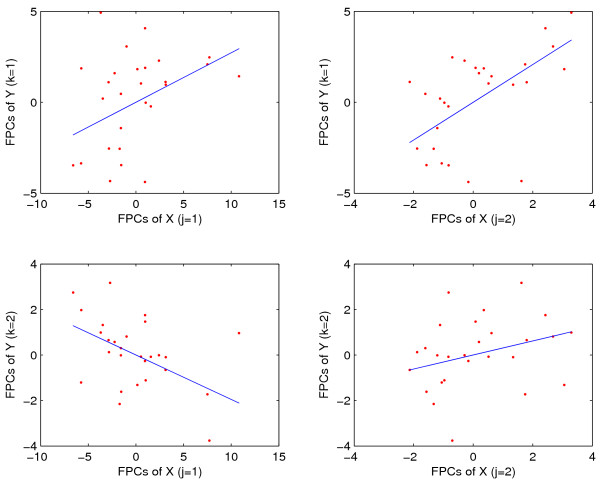
Scatterplots of response functional principal component scores *ξ*_*k *_versus predictor scores *ζ*_*j*_, for first two principal components, superimposed on simple linear regression lines without intercept, for strict maternal genes with embryo phase expressions as predictors *X *and pupa-adult phase expressions as responses *Y*. The functional coefficient of determination *R*^2 ^is 0.5503, with the bootstrap *p*-value *p *= 0.0020.

Decomposing the functional regression into the corresponding series of four simple linear regressions without intercept, the least squares fitted regression lines are shown in Fig. 8, along with the corresponding functional principal component scores. The positive relation of *ζ*_1 _versus *ξ*_1 _implies that embryonal expression that is above the mean trajectory, especially in the pattern delineated by the first embryonal eigenfunction, is associated with a pupa-adult response that is below the mean pupa-adult trajectory. This inverse pattern, with a slightly different emphasis on the nature of the deviations from the mean function, is also evident in the simple regressions of *ζ*_1 _vs *ξ*_2 _with its positive slope, and in the regression of *ζ*_2 _vs *ξ*_1 _with its negative slope. Here we note that a larger first principal component *ζ*_1 _corresponds to a response that is below the mean response, according to the negativity of the first eigenfunction of the response trajectories (the sign of the eigenfunctions is arbitrary). We note that (a) in order to evaluate the signs of the simple linear regression coefficients, they need to be considered in conjunction with the signs of the corresponding eigenfunctions; and (b) functional principal component scores measure the difference of a random trajectory and its mean trajectory, according to the Karhunen-Loève expansion (1), and therefore serve as proxies for the differences in gene expression between an individual's trajectory and the mean trajectory.

**Figure 9:**
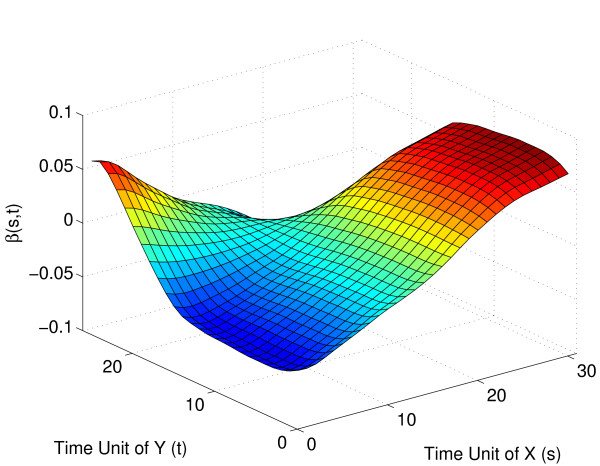
Estimated regression parameter function $\hat{\beta }$(*s*, *t*) for strict maternal genes with embryo phase as the predictor *X*(*s*) (plotted towards the right) and pupa-adult phase as the response *Y*(*t*) (plotted towards the left).

The inverse relation between embryonal and pupa-adult expression can also be visualized through the shape of the regression parameter surface (Fig. 9): Expression for the early part of the pupa-adult phase is larger for those trajectories with a larger increase in the embryonal expression over time (or, equivalently, those with a smaller decrease), however this effect is reversed for the later part of the pupa-adult expression. This reversal reflects the inverse functional relationship.

**Figure 10:**
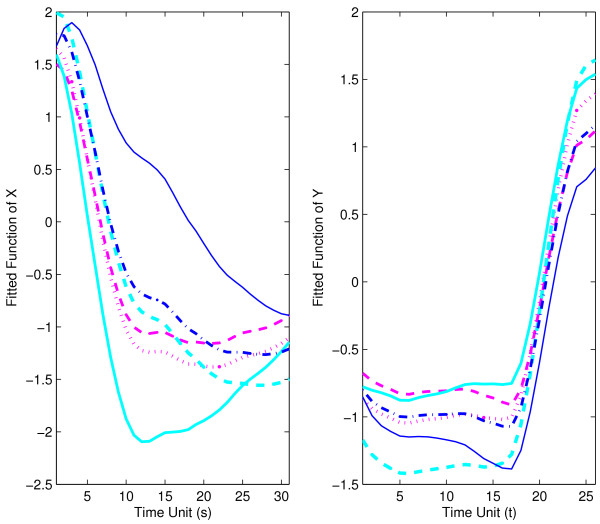
Predictor trajectories (left panel) and fitted response trajectories (right panel) for six randomly selected strict maternal genes.

A more direct way to interpret the functional regression is provided by looking at a few predictor trajectories and their associated responses (Fig. 10). A clear pattern that emerges is that predictor curves (in the left panel) that fall above the mean have associated response curves below the mean and vice versa, thus providing the graphical equivalent of an inverse functional relationship: Those genes characterized by increased expression during the embryonal period express less in the pupa-adult period. This observed negative association is of interest, as it is quite unexpected and pinpoints a dynamic interaction of life cycle stage and gene expression. The underlying mechanism causing this phenomenon and the strong differences between muscle and maternal genes is unknown and further studies to uncover it would be of interest.

**Figure 11:**
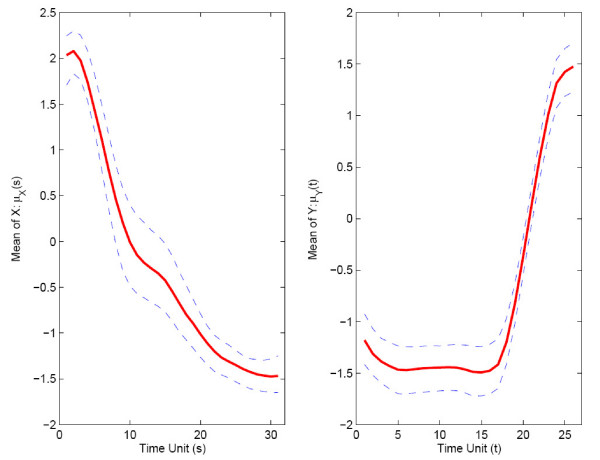
Estimated mean function with 95% bootstrap confidence intervals for strict maternal genes in embryo phase (for predictor *X*, left panel) and pupa-adult phase (for response *Y*, right panel), respectively.

**Figure 12:**
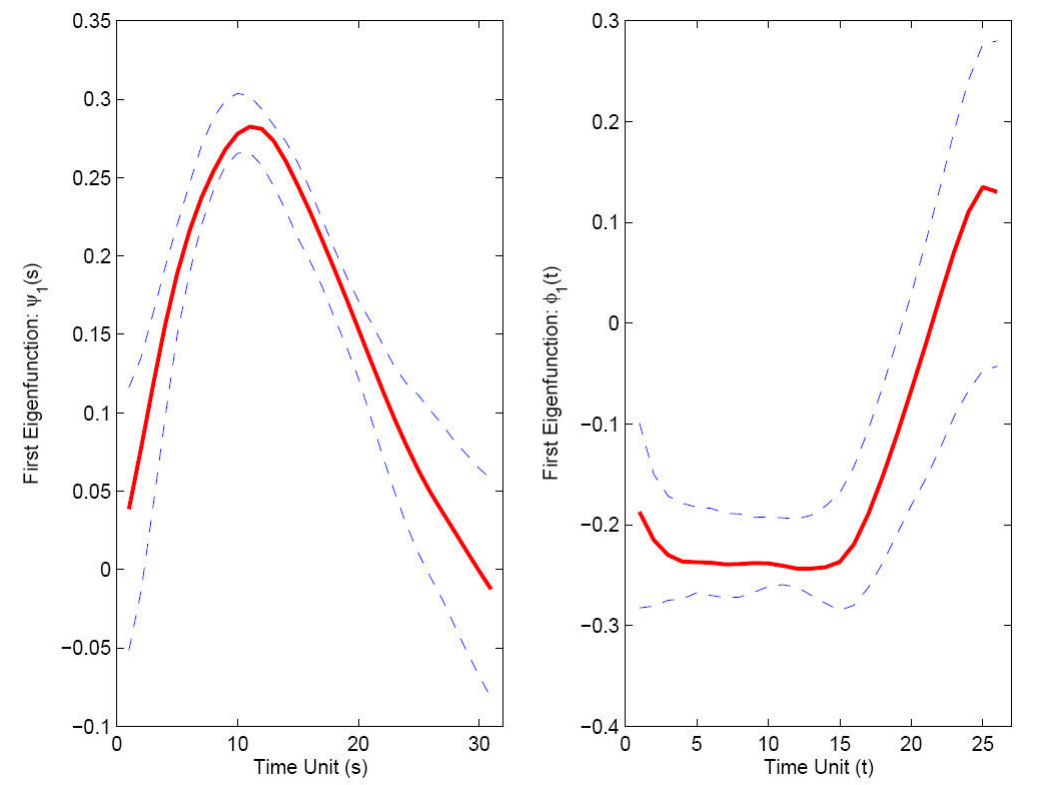
First estimated eigenfunctions with 95% bootstrap confidence intervals for strict maternal genes in embryo phase (predictor *X*, left panel) and pupa-adult phase (response *Y*, right panel).

**Figure 13:**
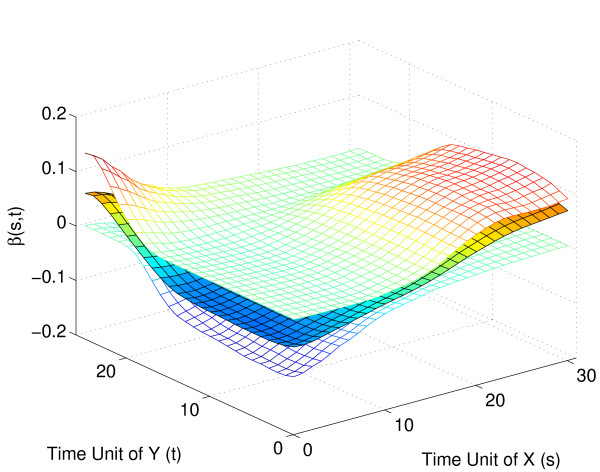
Estimated regression parameter function $\hat{\beta }$(*s*, *t*) with 95% bootstrap confidence intervals for strict maternal genes with embryo phase as the predictor *X*(*s*) (plotted towards the right) and pupa-adult phase as the response *Y*(*t*) (plotted towards the left).

For bootstrap inference (section 2.4), we construct *B *= 1000 bootstrap samples and also null bootstrap samples. For the choices of the number of included components *J *for predictor processes and *K *for response processes, implementing the criterion of 85% of variance explained, the relative frequencies of bootstrap selections were as follows: *J *= 1 in 33.7%, *J *= 2 in 66.3%, and *K *= 1 in 1.6%, *K *= 2 in 97.7%, and *K *= 3 in 0.7% of all bootstrap samples. Pointwise 95% bootstrap confidence intervals are shown along with the function estimates for mean functions *μ*_*X *_and *μ*_*Y *_in Fig. 11, and for the first eigenfunctions for predictor and response in Fig. 12. The 95% bootstrap confidence surfaces along with the estimated parameter function surface can be found in Fig. 13. In view of the large and dominant size of the area where the confidence surfaces do not include 0, it seems likely that the regression is significant.

To more unequivocally establish significance, we also ran the bootstrap test, described above, which is based on the functional coefficient *R*^2 ^as test statistic and the null bootstrap. This led to a *p*-value of *p *= 0.0020, for the functional coefficient of determination *R*^2 ^= 0.5503, providing evidence that the functional regression relation is significant here, with a sizable functional coefficient of determination.

## Discussion and Conclusion

### Comparison with simpler approaches

A natural question is whether the dimension reduction afforded by functional linear regression through regressing the functional principal components on each other is really useful for modeling gene expression trajectories and their relationships. To investigate this, we compared the proposed method with two simpler alternatives, both utilizing conventional linear regression models.

In the first alternative approach, we use the gene expression measurements of as many predictor time points as possible. This amounts to using the predictor expression at every second predictor trajectory time point to obtain the response at each time point where the response was recorded, fitting a multiple linear regression with 16 predictors. Note that one cannot use all 31 predictor time points in this classical regression approach, since then the number of predictors would be larger than the sample size which is not possible. This approach requires to fit as many such regressions as there are response time points, and therefore it requires a large number of parameters with the associated problems. So while this approach is conceptually and numerically simple, it does not provide dimension reduction, does not take into account the continuity of the underlying trajectories, and is subject to measurement errors in the predictors. This method is also not amenable to global significance analysis, as one encounters a multiple testing problem when dealing with the many separate regressions (and their associated coefficients of determination). In the following, we refer to this as the multiple regression approach.

In a second alternative approach, we first average all response measurements and then all predictor measurements to obtain a scalar response and associated scalar predictor. This corresponds to replacing all trajectories by constants. Then we run a simple linear regression of the response on the (single) predictor. This approach includes drastic dimension reduction but fails to model highly non-constant predictor and response trajectories as encountered for the *Drosophila *life cycle genes. This approach will be referred to as the simple regression model.

These methods are then compared in terms of their one-leave-out prediction errors for the muscle and the maternal gene groups: One removes one predictor-response trajectory pair at a time and then fits the corresponding approach (i.e., obtains the regression parameters for the sample that is reduced by the left out pair). Then the predictor data of the left out pair are fed into these fitted models and the resulting predicted responses at the times where the responses were obtained are recorded and compared with the actual observed responses. The differences are squared and averaged over the response times and then are averaged over the sample size of the gene group by recalculating them in turn for each left out pair. This procedure provides the averaged squared prediction error (SPE) and is a measure of the quality of the model and how well it can predict the actual observed data for a new data point.

The comparison of the above two alternative approaches with the functional regression method using this predictive quality measure led to the following squared one-leave-out prediction errors (SPE) as shown in Table 1. It emerges that functional linear regression provides a more sensible approach for the analysis of gene expression trajectories than classical regression approaches.

**Table 1:** Comparison of SPE using functional regression (FR), multivariate regression (MR), and simple regression (SR).

Gene group	FR	MR	SR
Muscle	1.03	2.17	2.89
Strict maternal	0.60	1.50	1.44

### Role of functional regression for gene expression trajectories

This paper explores functional regression approaches and their application to gene expression pro-files. Ultimately, useful statistical methodology should facilitate insights into the dynamics of microarray gene expression time courses. Our results in the application to temporal gene expression profiles for *Drosophila *life cycle indicate that while iterated reactivation of similar genes occurs throughout ontogenesis, there are gene-group specific differences in the reactivation patterns. Investigating the nature of these patterns eventually may provide a connection to the molecular basis of development. As a step towards this goal, we have proposed to quantitatively relate similar genetic pathways at different life stages. Functional regression is an adequate and promising tool for this purpose.

In general, a functional data analysis approach is well suited for the analysis of gene expression profiles, complementing the time series approaches that have been advocated for time course gene expression data [19,20]. We discuss in the following the pros and a few cons of the functional linear regression approach.

1. Easily handles a large variety of designs, including those where trajectories are sampled at many fixed times, or are sparsely sampled in random designs [13]. Totally at random missing observations present no problem (as long as two measurements per trajectory remain available).

2. Works under minimal assumptions, as it is a highly flexible nonparametric approach. The shapes of predictor and response trajectories can be arbitrary (only restriction is smoothness). No stationarity is required (as by some time series approaches).

3. Dimension reduction aspect avoids large number of parameters problem with increased variability and the need for multiple testing adjustments for valid inference. Trajectory dimension is chosen data-adaptively, enhancing the flexibility.

4. Decomposition into a series of simple linear regressions aids interpretation and motivates functional coefficient of determination *R*^2^, summarizing the strength of these regressions and representing an overall measure of the strength of the functional regression relation. It also serves as the bootstrap test statistic, discussed before.

5. Availability of associated graphical devices and visualization, characterizing the nature of the relationship between gene expression trajectories: These include (a) simple linear regressions through the origin of all response principal component scores on all predictor principal component scores, which in their entirety are equivalent to the functional linear regression; (b) the regression parameter surface *β*(·,·); and (c) the side-by-side plots of predictor functions and their fitted responses, that may be displayed for a subsample of the data and provide a trajectory-wise representation of the fitted model. These side-by-side plots allow for the easiest overall interpretation, while the simple linear regression lines also can be nicely interpreted when taking into account the shape of the relevant eigenfunctions. The regression parameter surface has the advantage to summarize the fitted model in one plot, and provides an interpretation of how the values of response trajectories at each fixed domain point depend on the entire predictor trajectories via a weight function.

6. Bootstrap as a useful inference tool to construct confidence bands for the functional components of the models such as mean and eigenfunctions and regression parameter surface, and to infer the overall significance of the functional regression relation. For the latter, the functional coefficient of determination *R*^2 ^is a natural target statistics. This allows the usual interpretation regarding the strength of a regression relationship, albeit here for relating random trajectories to each other.

On the con side, the interpretation of the regression parameter function is complex and requires careful scrutiny, as exemplified in the results. The decomposition into simple linear regressions can ameliorate this situation only to some extent. While the simple linear regressions themselves are easy to interpret, in the context of the functional regression model one must take into account the shape of the respective eigenfunctions.

The computational effort is relatively high, compared to classical regression approaches. The PACE package includes default features to accelerate computations for a large number of genes, including prebinning the measurements. Besides this package, there are not many software options available at this point which implement the described approach.

### Insights for life cycle gene expression

Using the functional regression tool, a first finding is that for the *Drosophila *life cycle, later life gene expression is significantly related to early life gene expression across genes. A second finding is that the nature of the relationship of later phase gene expression profiles with the profiles of earlier phases of the life cycle is specific to a gene group. For the group of muscle-specific genes, increased expressions along the mean trajectories for the embryonal phase are coupled with equally increased expression along the mean expression for the pupa-adult phase. We refer to this kind of relationship as functional proportionality, implying that similar relative expression levels for muscle-specific genes occur during both embryonal and adult phases. Such simple positive coupling of gene expression trajectories points towards close ties between the expressions at different stages and can be interpreted as positive developmental correlation.

While myogenesis in embryonal and adult phases are different processes of somatic muscle formation, they share common biological processes, such as myoblast fusion and neuromuscular junction [3]. Among the 23 "muscle" genes, unsurprisingly, seven (including CG11914, CG9480, Mlc1, CG8154, Upheld, MSP-300, and Paramyosin) are involved in mesoderm development [21]. The expression of those genes during adult muscle development indicates that their function might extend to the pupal stage. Chen and Olson pointed out that many in vitro studies have implicated several classes of proteins in myoblast fusion, including cell-adhesion molecules, protein kinases and phospholipases [22]. Six genes are related to cell adhesion/receptor activity/signal transduction/protein kinase/phosphate transport (including CG10483, Dscam, CG7028, CG9098, CG9090, and CG18020) [21]. Most research on molecular pathways on myoblast fusion in *Drosophila *has focused on the embryonal phase, however it is conceivable that similar molecular mechanisms might be involved in adult satellite-cell fusion analogous to myoblast fusion during embryogenesis [22]. It is also noteworthy that six genes (including Mlc1, Mhc, Upheld, MSP-300, Paramysosin and CG1826) are related to myofibril assembly/actin assembly or binding/microfilament motor activity, and five genes (Dscam, Slowpoke, CG9098, CG7565 and CG8256) showed neuronal expression. Dscam is involved in axon guidance, neuron development and peripheral nervous system development, and Slowpoke in synaptic transmission. These genes might be involved in neuromuscular junction building during muscle development. There is also good evidence that during pupal development, motorneuronal innervation is critical to the specification of at least one set of muscle fibers [4,21,23].

Identification of the muscle gene cluster was based on evidence of the involvement of the same genes in the two phases as suggested in Arbeitman's study [4]. Our results further show that beyond the involvement of these genes in both phases also their levels of expression are a characteristic that is relevant for normal development. Although this is plausible, no previous systematic study that we are aware of has provided evidence for this stability in expression level for this class of genes. While most studies have been focusing on certain genes, or on mean levels of expression for groups of genes, an advantage of our approach is that we study a set of genes as a system, focussing on association of their expression profiles over the life cycle.

In the case of maternal genes, we also find a strong functional relationship between the life cycle phase expressions, but their association is of a quite different nature than for the group of muscle genes. We detected a negative coupling in the sense that predictor trajectories above the overall mean of predictors are associated with response trajectories below the overall mean of responses. More specifically, the existence of an initial rapid decline and deep trough in the middle of embryonal gene expression is associated with larger pupa-adult expression throughout, and if there is an initial larger embryonal expression with a more modest decline then this is associated with relatively low pupa-adult expression, including a deeper trough in the response trajectories. This type of functional relationship is surprising and can be described as inverse functional proportionality. It points to a negative developmental correlation, and to the best of our knowledge, such a negative association of gene expression between different phases of development has not been previously reported. It will be of interest to discover the underlying mechanisms, as not much is known about these genes.

Gaining biological knowledge by observing and analyzing developmental time courses of gene expression in simpler organisms (with their short life spans) paves the way to gain knowledge of the corresponding genetic mechanisms in more complex organisms. For instance, both embryonic and pupal muscle development in Drosophila shows striking similarities with elements of pattern formation in vertebrate muscle. The presence of both positive and negative coupling of expression patterns is intriguing and points to complex regulatory mechanisms.

## Authors' contributions

HGM initiated the project and contributed basic ideas and writing. JMC contributed statistical modelling, implementation and microarray data analysis. XL provided biological interpretation and contributed to the writing.

## Appendix: Mathematical Details and Derivations

For square integrable predictor and response processes *X*(*s*) resp. *Y*(*t*) with domains S and T, mean functions *μ*_*X*_(*s*) = *E*(*X*(*s*)), *μ*_*Y *_(*t*) = *E*(*Y*(*t*)) and auto-covariance functions *G*_*X*_(*s*_1_, *s*_2_) = cov(*X*(*s*_1_), *X*(*s*_2_)), *G*_*Y *_(*t*_1_, *t*_2_) = cov(*Y*(*t*_1_), *Y*(*t*_2_)), one defines the auto-covariance operators

$$\begin{array}{ll} (A_{G_{X}}f)(t)=\int f(s)G_{X}(s,t)ds, & (A_{G_{Y}}f)(t)=\int f(s)G_{Y}(s,t)ds. \end{array}$$

These are linear operators in the Hilbert space *L*^2 ^of square integrable functions, with eigenfunctions characterized as solutions of the eigen-equations (*Af*)(*t*) = *λf*(*t*), where *λ *is an eigenvalue.

We denote by *φ*_*j*_(*s*) and *ψ*_*k*_(*t*) the sequences of orthonormal eigenfunctions for *X *and *Y*, with non-increasing eigenvalues *λ*_*j *_and *τ*_*k*_, ∑_*j*_*λ*_*j *_< ∞ and ∑_*k*_*ξ*_*k *_< ∞, satisfying

(9) $$\begin{array}{llll} G_{X}(s_{1},s_{2})=\sum_{j}\lambda_{j}\varphi_{j}(s_{1})\varphi_{j}(s_{2}), & s_{1},s_{2}\in S, & G_{Y}(t_{1},t_{2})=\sum_{k}\tau_{k}\psi_{k}(t_{1})\psi_{k}(t_{2}), & t_{1},t_{2}\in T. \end{array}$$

The random coefficients $\xi_{j}=\int_{S}(X(s)-\mu_{X}(s))\varphi_{j}(s)ds$ and $\zeta_{k}=\int_{T}(Y(t)-\mu_{Y}(t))\psi_{k}(t)dt$ are uncorrelated random variables, respectively, with means *E*(*ξ*_*j*_) = 0, *E*(*ζ*_*k*_) = 0, and variances var(*ξ*_*j*_) = *λ*_*j*_, var(*ζ*_*k*_) = *τ*_*k*_. The eigenfunctions *ψ*_*k*_, *φ*_*j *_are also referred to as principal component functions and the random effects *ξ*_*j*_, *ζ*_*k *_as functional principal component scores. A further assumption is that all functions {*μ*_*Y*_, *ψ*_*k*_}, {*μ*_*X*_, *φ*_*j*_} are smooth, usually it is assumed they are twice continuously differentiable.

*Derivation of (5). *Replacing function *β*(*s*, *t*) on the r.h.s. of (3) by the representation (4) and *X *- *μ*_*X *_by the Karhunen-Loève expansion (1), one finds, using the orthonormality of the eigenfunctions *φ*_*j*_, that

$$E(Y(t)|X)=\mu_{Y}(t)+\sum_{k=1}^{\infty }\sum_{j=1}^{\infty }\beta_{kj}\xi_{j}\psi_{k}(t).$$

Then (2) and the orthonormality of eigenfunctions *ψ*_*k *_imply

(10) $$E(\zeta_{k}|X)=\int (E(Y(t)|X)-\mu_{Y}(t))\psi_{k}(t)dt=\sum_{j=1}^{\infty }\beta_{kj}\xi_{j},$$

for all *k *≥ 1. Since {*ξ*_*j*_, *j *= 1, 2,...} are uncorrelated random variables, it follows from model (10) that

(11) $$E(\zeta_{k}|\xi_{j})=E[E(\zeta_{k}|\xi_{1},\xi_{2},...)|\xi_{j}]=E[E(\zeta_{k}|X)|\xi_{j}]=E\left[\sum_{j=1}^{\infty }\beta_{kj}\xi_{j}|\xi_{j}\right]=\beta_{kj}\xi_{j},$$

for all pairs *k*, *j*, and (5) follows.

*Regression parameter function β*(*s*, *t*) *determines the slopes β*_*kj*_. Using representation (4) of *β*(*s*, *t*), and the orthonormality properties of eigenfunctions *φ*_*j *_and *ψ*_*k*_, one finds

(12) ∫ ∫ *β*(*s*, *t*) *φ*_*j*_(*s*) *ψ*_*k*_(*t*) *dt *= *β*_*kj*_   for all *j*, *k*.

Therefore, the function *β*(*s*, *t*) determines all slope coefficients *β*_*kj *_uniquely.

*Functional R*^2^. One can show

(13) $$R^{2}=\frac{\int_{T}\mathrm{var}(E[Y(t)|x])dt}{\int_{T}\mathrm{var}(Y(t))dt}=\sum_{j=1}^{\infty }\frac{\sum_{k=1}^{\infty }R_{kj}^{2}\tau_{k}}{\sum_{k=1}^{\infty }\tau_{k}},$$

where

(14) $$R_{kj}^{2}=\frac{{\left(E(\xi_{j}\zeta_{k})\right)}^{2}}{E\xi_{j}^{2}E\zeta_{k}^{2}}=\frac{{\left[\mathrm{cov}(\xi_{j},\zeta_{k})\right]}^{2}}{\lambda_{j}\tau_{k}}$$

are the coefficients of determination for the simple linear regressions of *ξ*_*k *_on *ζ*_*j*_. This means that in the decomposition approach, the overall functional coefficient of determination *R*^2 ^is obtained as a sum (summing over the contributions of all functional principal components in the predictor set) of eigenvalue-weighted averages of the coefficients of determination of the simple linear regressions (averaging over the contributions of all functional principal components in the response).
